# The Initial Part of Polymorphic Ventricular Tachycardia as a Clue for the Sustainability of Tachycardia and Ablation Success: A Varying Degree of Purkinje–Myocardial Complicity?

**DOI:** 10.19102/icrm.2023.14066

**Published:** 2023-06-15

**Authors:** Duygu Kocyigit Burunkaya, Ozcan Ozeke, Ahmet Korkmaz, Firat Ozcan, Meryem Kara, Elif Hande Ozcan Cetin, Mehmet Yaman, Can Demirhan, Abdullah Tuncez, Umuttan Dogan, Osman Can Yontar, Serkan Cay, Dursun Aras, Serkan Topaloglu

**Affiliations:** ^1^Department of Cardiology, University of Health Sciences, Ankara City Hospital, Ankara, Turkey; ^2^Department of Cardiology, Eregli Echomar Hospital, Zonguldak, Turkey; ^3^Department of Cardiology, Selcuk University, Konya, Turkey; ^4^Department of Cardiology, Akdeniz University, Antalya, Turkey; ^5^Department of Cardiology, University of Health Sciences, Samsun Education and Research Hospital, Samsun, Turkey; ^6^Department of Cardiology, Istanbul Medipol University, Istanbul, Turkey

**Keywords:** Multiple exits, myocardial substrate, pleomorphic ventricular tachycardia, polymorphic ventricular tachycardia, Purkinje

## Abstract

The cardiac Purkinje system is capable of very rapid burst activity suggestive of its potential role in being a driver of polymorphic ventricular tachycardia (VT) (PMVT) or ventricular fibrillation (VF). It plays a pivotal role, however, not only in the triggering of but also the perpetuation of ventricular arrhythmias. A varying degree of Purkinje–myocardial complicity has been blamed in determining not only the sustained or non-sustained nature of PMVT but also the pleomorphism of the non-sustained runs. The initial part of PMVT before cascading to the whole ventricle to establish disorganized VF can give important clues for ablation of PMVT and VF. We present a case of an electrical storm after acute myocardial infarction that was successfully ablated after identifying Purkinje potentials that triggered polymorphic, monomorphic, and pleiomorphic VTs and VF.

## Case presentation

A 63-year-old man with ischemic cardiomyopathy presented with an electrical storm (ES) and recurrent defibrillator shocks. He had experienced an anterior myocardial infarction (MI) 3 years earlier that caused his left ventricular ejection fraction to decrease to 30%; subsequently, an implantable cardioverter-defibrillator (ICD) was implanted for primary prophylaxis. On admission, emergency cardiac catheterization was performed in a different center, which revealed 98% stenosis of the proximal left anterior descending coronary artery with chronic total occlusion of the right coronary artery. Despite successful percutaneous revascularization, the ES failed to respond to medical therapy with amiodarone, lidocaine, β-blockade, and β-agonists, and the patient was referred for an emergency ablation for his refractory polymorphic ventricular tachycardia (VT) (PMVT) with repetitive ICD shocks. Cardiopulmonary resuscitation was applied during patient transfer in the ambulance, and the patient was intubated at a local hospital during transit.

**Figure 1:**
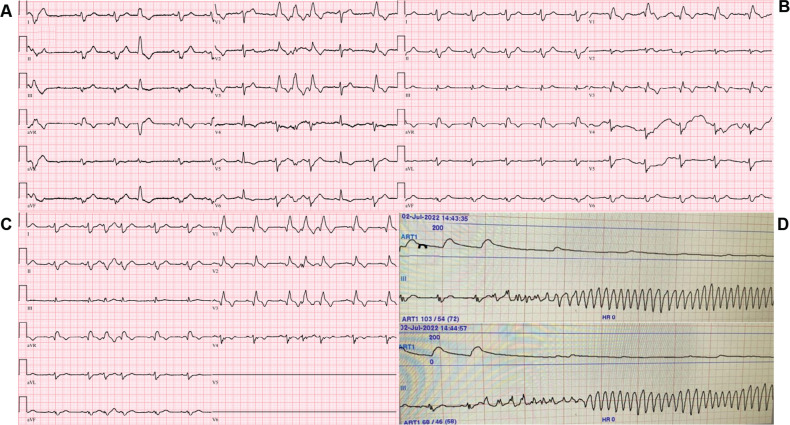
Twelve-lead electrocardiograms taken at arrival **(A)** and the following day **(B and C)** are seen. **D:** Note the first several cycles of episodes before the development of the sustained polymorphic ventricular tachycardia, which, although polymorphic, may be nearly identical at different times on telemetry strips.

**Figure 2:**
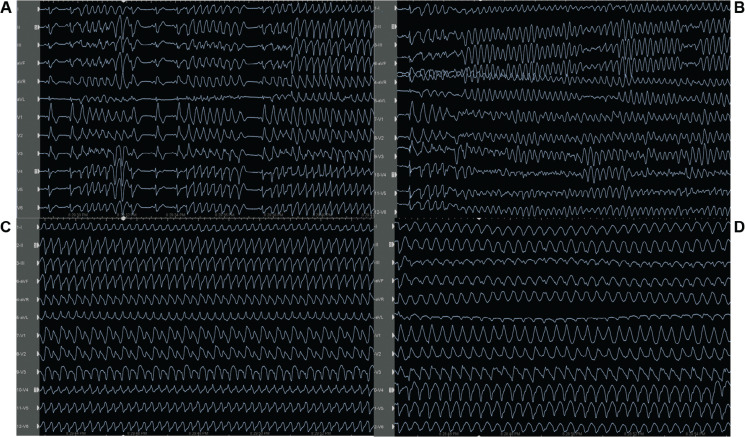
Electrocardiograms recorded during the electrophysiology study show several episodes of non-sustained polymorphic **(A)**, sustained polymorphic **(B)**, pleomorphic **(C)** note the alternans changes in precordial leads), and monomorphic **(D)** ventricular tachycardia.

**Figure 3:**
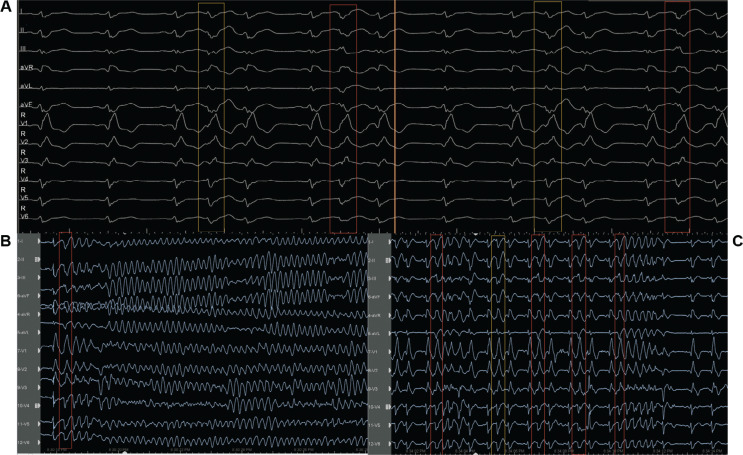
**A–C:** Electrocardiograms recorded during the electrophysiology study. Note that the initiating first several cycles of episodes before the development of the sustained polymorphic ventricular tachycardia, although polymorphic, may be nearly identical to the preceding isolated premature beat (morphology 1, red rectangle with negative polarity in aVL; morphology 2, yellow rectangle with positive polarity in aVL).

**Figure 4:**
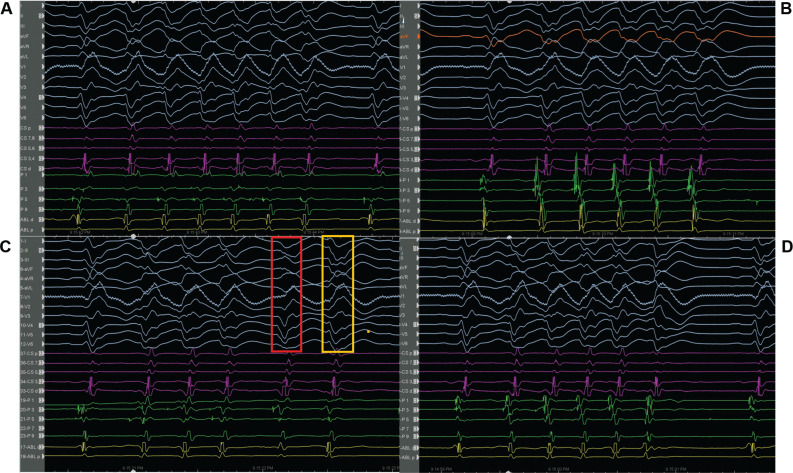
The high-frequency Purkinje potentials preceding the QRS at an early ventricular activation site with 1-to-1 Purkinje activity **(A and C)** or a dissociated form **(B)**. The morphology of the ventricular complexes, originating from the Purkinje fibers, is changing (note the QRS changes in aVL in **A–D**), suggesting different Purkinje–muscle outbreak terminations of polymorphic ventricular tachycardia preceded by the slowing **(B and C)** or disappearance **(D)** of Purkinje potentials **(C)**. **C:** Note also a pair of morphologically distinct premature beats with different conduction times to local muscle (morphology 1, red rectangle with negative polarity in aVL; morphology 2, yellow rectangle with positive polarity in aVL).

**Figure 5:**
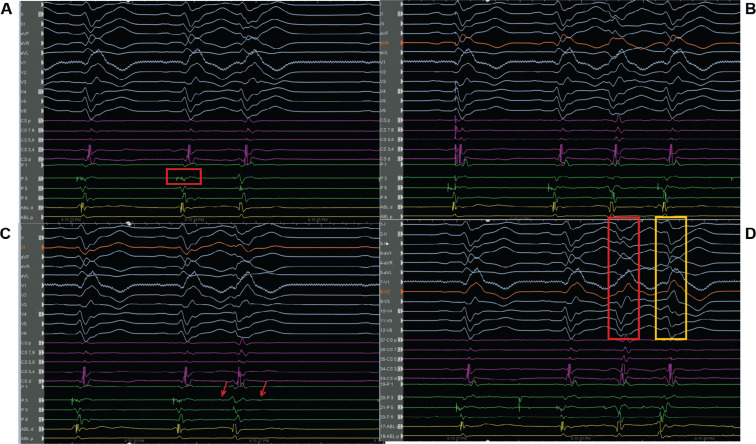
The high-frequency Purkinje potentials preceding the QRS at an early ventricular activation site also precede activation during sinus rhythm. During sinus rhythm, fused Purkinje potentials are seen before the onset of the QRS **(A)**. Premature beats originating from the left ventricular Purkinje system are seen as isolated **(A)** and interpolated **(B)** forms. The second Purkinje discharge is not conducted to the ventricle due to exit block (**C**; note red arrows). **D:** Note each QRS complex is morphologically different but preceded by a Purkinje potential with a varying conduction time. Note the mild changes in QRS morphology between the red and yellow rectangles (morphology 1, red rectangle with negative polarity in aVL; morphology 2, yellow rectangle with positive polarity in aVL).

**Figure 6:**
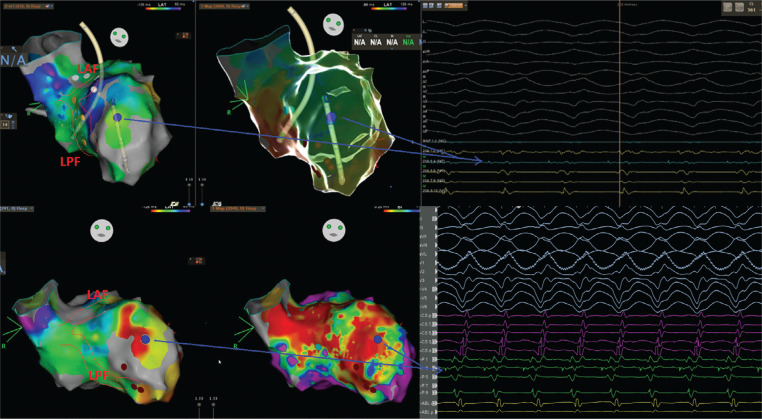
The 3-dimensional electroanatomic mapping shows the conduction system (yellow and red dots) and the Purkinje potential (blue dot) during the monomorphic ventricular tachycardia. The changes in ventricular cycles are preceded by a similar change in Purkinje cycles. *Abbreviations:* LAF, left anterior fascicle; LPF, left posterior fascicle.

**Figure 7:**
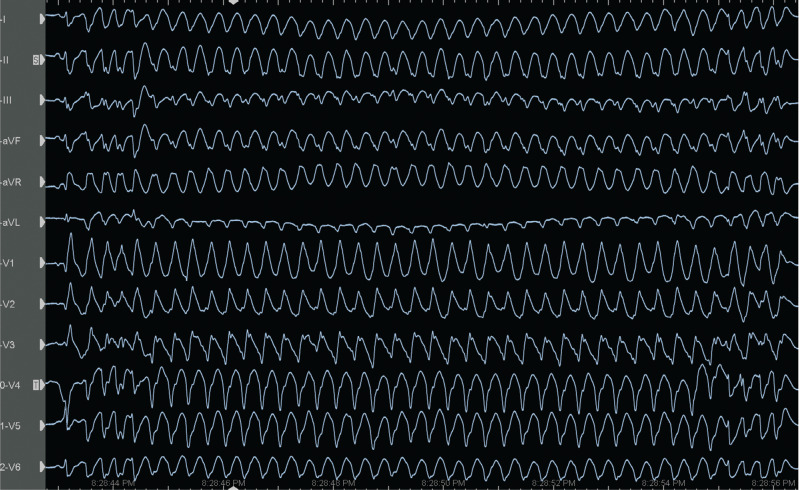
Both initiation and termination of the monomorphic ventricular tachycardia were determined by similar non-sustained ventricular cycles.

On admission to the coronary care unit on Friday night, the patient had an acute kidney injury and suspected hypoxic brain injury. The patient’s 12-lead electrocardiogram (ECG) showed sinus rhythm with intermittent right bundle branch block (RBBB) and runs of premature ventricular complexes (PVCs) with the morphology of an RBBB **(Figure 1A)**; therefore, we decided to follow the patient in the coronary care unit. The following Saturday morning, a persistent RBBB developed on ECG **(Figure 1B)**, and the same RBBB morphology ran similar to the appearance of sinus beats **(Figure 1C)**; he again suffered recurrent PMVTs requiring ICD shocks on Saturday evening. Interestingly, telemetry displayed the first several cycles of episodes before the development of the sustained PMVT, which, although polymorphic, were nearly identical **(Figure 1D)**. This suggested a consistent pattern of activation among these cycles until the rhythm became more disorganized; at this time, an emergency electrophysiological study on Saturday evening was performed using a pair of decapolar catheters positioned in the right and left ventricles. While setting up for transseptal catheterization to access the left ventricle, several episodes of non-sustained **(Figure 2A)** and sustained **(Figure 2B)** PMVT continued, requiring ICD shocks. Similar to telemetry tracings **(Figure 1D)**, the first several cycles of episodes before the development of the sustained PMVT, although polymorphic, were nearly identical to the non-sustained ones **(Figure 3)**. Fortunately, the ES was converted to non-sustained PMVT forms and also more hemodynamically stable pleomorphic **(Figure 2C)** and monomorphic VT (MMVT) **(Figure 2D)** during mapping of the septal distal Purkinje network (PN) (bump mapping). The high-frequency Purkinje potentials (PPs) preceding the QRS at an early ventricular activation site were found at the inferoseptal aspect of the left ventricle with discrete morphological variations **(Figures 4–6)**, suggesting repetitive focal activity or re-entry into the PS.^1^ Interestingly, this initial His–Purkinje system (PS) (HPS) activity was detected before both PMVT and MMVT **(Figure 7)**.

For Purkinje–VTs, critical participation of the HPS in VT was defined by a His, left, or right bundle or fascicle potential closely associated with the VT QRS, with passive retrograde activation excluded by any of the following: (1) variation in the interval between His-bundle (or fascicular) potentials preceding and predicting V–V interval variations, (2) demonstration of an antegrade activation sequence of a bundle branch (or fascicle) during tachycardia, or (3) abolition of the VT by bundle branch (or fascicle) ablation.^2,3^ What is the mechanism of these abnormal wobbling and pleomorphisms seen in **Figure 4** during non-sustained PMVT episodes?

## Discussion

The cardiac PS is capable of very rapid burst activity suggestive of its potential role in being a driver of PMVT or ventricular fibrillation (VF).^1^ Recently, there has been a growing body of evidence that the PN plays a pivotal role not only in the triggering but also in the perpetuation of ventricular arrhythmias.^1,4–9^ They can manifest as both MMVT and PMVT in patients with normal hearts as well as in patients with structural heart diseases or channelopathy.^10–12^ The PS may trigger or maintain these arrhythmias by automaticity, re-entry, or triggered activity during multiple conditions such as electrolyte imbalance, catecholamine or other drug exposure, and acute/subacute MI, during which Purkinje fibers (PFs) can survive within the border zone of scar.^13–19^ In general, the MMVT in patients with a previous history of MI is commonly caused by re-entry circuits in the myocardial scar area or its borders.^20^ However, in certain cases, VTs after MI have re-entrant circuits mediated by the PF; examples include bundle branch re-entry (BBRT), inter- or intrafascicular re-entry, and focal Purkinje VT. Despite successful revascularization,^15^ the surviving subendocardial PF localized to the border zone of the MI (specifically in the left posterior fascicle)^17,21^ might trigger rapid, repetitive depolarizations that can occur spontaneously or could be induced by PVCs or premature Purkinje-related complexes (PPCs).^13,16,17^

An initial sharp potential preceding the larger and slower ventricular electrogram during sinus rhythm represents a distal Purkinje component, whereas longer intervals indicates proximal Purkinje fascicle activation.^1^ High-frequency sharp potentials consistent with a possible Purkinje origin **(Figures 4–6)**, similar to the preceding target PVCs/PPCs **(Figure 1C)**, were identified in an area that would correspond to the location of Purkinje ramifications of the left posterior fascicle along the scar border zone **(Video 1)**.^18,22^ High-frequency sharp Purkinje potentials were also identified in an area that would correspond with the location of the left anterior fascicle **(Video 2)**. While an early ventricular activation site was found at the inferoapical septal aspect of the left ventricle, the proximal septal region was found to be activated late **(Figure 6, Video 1)**.

Because a conduction disease is expected to be present in the His-bundle branches in the setting of BBRT, a similar or greater conduction impairment should likely be present for distal fascicular re-entry. Herein, studies suggest the existence of re-entry involving the Purkinje–myocardial junction (PMJ), at least in the early stages of VF.^14^ Therefore, a varying degree of Purkinje–myocardial complicity has been blamed in determining not only the sustained or non-sustained nature of PMVT but also the pleomorphism of the non-sustained runs.^8,20,23,24^ The absence of late potentials after effective ablation at sites with PPs argues against re-entry confined to the PFs alone.^3^ The critical conditions of re-entry dynamics regarding the heterogeneity of the PMJ were shown by Gilmour and Watanabe in various experiments on canine preparations.^25^ In addition, Lazzara et al. showed that the (proximal) bundle branches were the preferential sites for conduction block during premature stimulation, whereas conduction could still occur through some distal fibers (interfascicular fibers of the left bundle branch and septal fibers of the right bundle branch).^26^ These Purkinje triggers can cause a single isolated **(Figure 5A)**, interpolated **(Figure 5B)**, or single beat with a second Purkinje discharge that is not conducted to the ventricle (**Figure 5C**, exit block; note the red arrows), or a pair of morphologically distinct premature beats with different conduction times to local muscle (**Figures 3 and 5D**; note the QRS changes in the aVL lead between the yellow and red rectangles).^27^ Both initial Purkinje activations were different, indicating an involvement of a different part of the peripheral PS **(Figure 4C)**. During sinus rhythm, the PPs closely preceded the ventricular muscle activity, indicating peripheral arborization **(Figures 4 and 5)**.^4^ However, differing conduction times were associated with varying morphologies, suggesting either changes in the ventricular activation route or origin from another part of the PS **(Figure 4C)**.^1^ For arrhythmias of Purkinje origin, therefore, pace-mapping is exceedingly difficult to execute.^28^ These repetitive beats can trigger non-sustained **(Figure 2A)** or sustained PMVT **(Figure 2B)** or MMVT **(Figures 2D and 5B)**.

The PS is a requirement for PMVT at some initial stage, but the excitation pattern evolves into a state in which PFs are not needed for arrhythmia maintenance.^14^ While the PS provides the required structure for initial maintenance of the re-entry, once the re-entry becomes sustained, the PS allows for drift and the eventual establishment of intramyocardial re-entry.^14^ When these conditions are met, the PS becomes a bystander, with its activity being enslaved by the rotating activity in the myocardium.^14^ Haïssaguerre et al. reported that the activities maintaining VF are mainly generated from the PN and structural substrate, before spreading to establish disorganized VF.^8^ The initial ~5 s of VF is sustained by activities originating from localized substrate areas and the PN before “cascading” to the whole ventricle to establish disorganized VF **(Figure 3)**.^8,9,14,15^ The transition to disorganized VF was associated with the acceleration of initial re-entrant activities.^8^ Furthermore, at the initiation of the PMVT, the intervals between the PPs were gradually shortened, and thereby an intra-Purkinje block occurred.^29^ In their computerized 3-dimensional (3D) model of ventricles, Berenfeld and Jalife observed that re-entry was terminated if the PS was disconnected from the muscle before it reached a relatively steady state.^14^ Therefore, the arrhythmia termination is usually preceded by slowing or cessation of Purkinje activity **(Figures 4B and 4D)**.^8^ At this point, such a potential preceding ventricular activation during premature beats indicates that the latter originated from the PS, whereas its absence at the site of earliest activation may also indicate an origin from ventricular muscle or multiple Purkinje exits^1,4–6^ (**Figure 3**; note the mild QRS changes between the rectangles). It is also possible that linear catheters may have poor contact when recording the PPs; however, it was a reproducible finding during the study **(Figures 4B and 4D)**. The issue of multiple foci versus differing activation routes from limited foci remains unresolved in the absence of appropriate mapping coverage.^1^ Therefore, there is the possibility that not only the elimination of the triggering PVC/PPC but also the conduction block in the PN can suppress the triggering PVC/PPC and PMVT/VF.

The mechanism of pleomorphic VT **(Figure 2C)** or MMVT **(Figure 2D)** is eclectic but generally believed to be due either to re-entry using the fascicles and/or bundle branches and/or the myocardium, or possibly focal automaticity of Purkinje tissue^10^ after scar organization occurring concurrently with or after VF ablation.^30^ The presence of a PP during MMVT was validated by the presence of a PP during sinus rhythm at the same site^3^ **(Figure 6)**. The changes in ventricular cycles were constantly preceded by a similar change in Purkinje cycles in the MMVT form **(Figure 6)**. It is difficult to distinguish verapamil-sensitive fascicular VT from focal Purkinje VT using a 12-lead ECG; however, focal Purkinje VT is not responsive to verapamil.^31^ Indeed, the PPs alone are not a specific indicator of participation within the re-entry circuit.^3^ Re-entry using the PS has been well described in MMVTs such as BBRT and intra- or interfascicular re-entry.^31^ However, re-entry can also occur at the PMJ, resulting in VF initiation.^22^ The presence of both a PP and concealed entrainment during VT is required to indicate the involvement of the PFs in the re-entry circuit.^3^ Attempts at ventricular entrainment at these sites during MMVT resulted in PMVT in the current case. Polymorphic QRS excludes BBRT.

Catheter ablation targeting the PP responsible for triggering VF is possible and efficacious in several conditions, including idiopathic VF (a short-coupled variant of torsade de pointes), ischemic VF, and chronic myocarditis.^1,8,9,22,32^ The optimal time for ablation is often at the time of an ES. The most important issue before the ablation session is the recording of a 12-lead ECG of the triggering event, which can prove invaluable in regionalizing the origin of the triggering PVC/PHC or runs for more detailed mapping.^18^ PPCs have a narrower QRS duration, particularly when they originate from the left PS (<120 ms), where they exhibit an RBBB morphology. PPCs from the right PN have a left bundle branch block morphology and a wider QRS duration (130–150 ms) but an initially rapid deflection. The narrower left PPCs are probably due to the dense left arborization, allowing simultaneous capture of a greater part of the left ventricle **(Video 1)**.^9^ The suggested target for ablation of a focal Purkinje tachycardia is the earliest PP preceding the QRS in ventricular premature beats/VT.^1,31,33^ Patients with ischemic cardiomyopathy often present with >1 ectopic focus, and the risk of early recurrence of ES after successful ablation of a single trigger supports the strategy to ablate all ectopic foci.^34^ However, the potential may sometimes be seen to occur with intra-Purkinje block to the myocardium and not produce a PVC/PPC. The modification of the PN might be applied when the earliest site cannot be determined or is located close to the His bundle.^18^ It should also be kept in mind that the catheter manipulation sometimes produces transient bundle branch block by bumping the ectopic focus with the catheter. As a result, peripheral PPs no longer precede the local ventricular activation in sinus rhythm, and it makes mapping of the PN difficult.^18^

On the other side, bump mapping of the possible culprit PS may relieve the ES and allow mapping of the initial part of the non-sustained PMVT as a target, as seen in the current case. A new-onset RBBB had already developed in the current case **(Figure 1)** during hospitalization as a sign of the conduction disturbance and/or the primary or secondary characteristic of the ventricular arrhythmias.^35^ Pace-mapping may simultaneously capture the local myocardium as well as the local fascicles and PN.^23^ Thus, pacing at a site that represents the arrhythmogenic substrate may show a very different QRS morphology than the triggering PVC (because of local myocardial activation), while possible sole capture of a downstream fascicle (distal to the site of origin) may mimic fairly closely the clinical PVC yet not represent a site of culprit arrhythmogenicity.^20,23,28^ More important than in idiopathic VF, the use of an electroanatomic mapping system is recommendable. This allows both 3D reconstructions of the left ventricular endocardial surface with annotation of the location of the conduction system and delineation of myocardial necrosis or scar as low-voltage areas **(Figure 6)**.^34^ Besides annotation of the earliest activation during the ectopy, it allows display of the extent of myocardial necrosis and/or scar, and the catheter ablation may address more Purkinje tissue along the margin of the affected tissue.^34^ Indeed, the ablation does not need to be transmural because of the relatively superficial presence of the subendocardial PN.^28^

**Figure 8:**
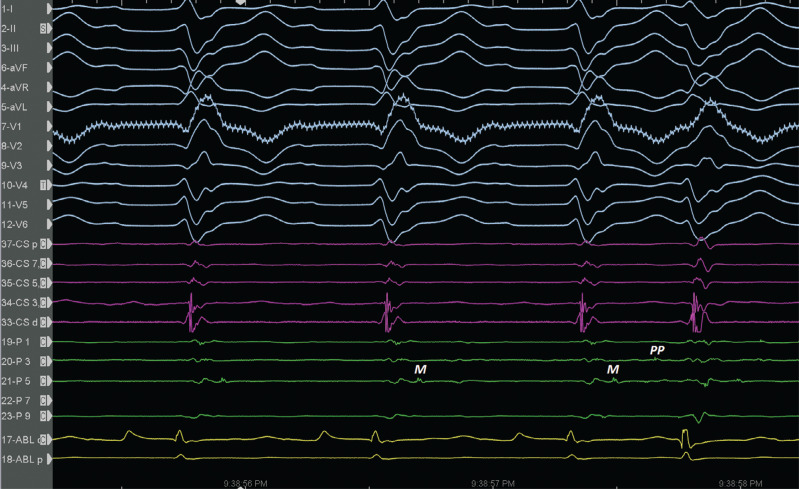
It is important to distinguish between a fascicular Purkinje potential from isolated muscle potentials. *Abbreviations:* M, local abnormal ventricular activity; PP, Purkinje potential.

On the other side, the PN in idiopathic VF is usually healthy; however, this differs from ischemic VF, in which the ventricular myocardium at the culprit PN usually has a low voltage with prolonged duration and fractionated late potentials **(Figures 5A and 8)** and is located near a scar border.^18^ These fractionated potentials **(Figures 5A and 8)** and the earliest activity-preceding beat **(Figure 6)** are found in the distal left posterior fascicle, with different activation sequences associated with both different electrocardiography morphologies **(Figure 5D)**. Therefore, it is important to distinguish between a fascicular potential from an isolated muscle potential **(Figure 8)**,^11,12^ stressing the importance of multielectrode Purkinje recordings for Purkinje recognition within fractionated myocardial electrograms **(Figure 5A)**. However, the key is to position the catheter over the fascicle in question and record the anterograde activation pattern in sinus rhythm **(Figure 5A)** and the arrhythmia.^11^ The absence of spontaneous PVCs/PPCs and the non-inducibility of PMVT are clinical endpoints of ablation, but these are not always practical. Surrogate endpoints include the abolition of local PPs and a slight delay in the occurrence of the local ventricular electrogram at the site of ablation during sinus rhythm.^1^ However, dissociated firing from the PN is sometimes seen after a successful ablation^18^; therefore, the demonstration of diastolic PPs indicating intra-Purkinje conduction block is another potential endpoint.^31^

Suppression of VF can be achieved not only by the elimination of triggering PVCs/PPCs but also by substrate modification of possible re-entry circuits in the PN to address multiple foci or local re-entry.^10,18,28^ Ablation of the surrounding PS without elimination of the culprit PP may also be sufficient to prevent VF recurrence.^36^ However, catheter ablation of the triggered ventricular premature complex is challenging when the earliest activation site cannot be identified or is located close to the His bundle. Therefore, a substrate modification of the PN should be considered.^18,28,37^ Ablation lesions in the current case were applied all along the length of the border zone to eliminate all detected PPs and local abnormal ventricular activities confined to the left interventricular septum. No arrhythmia recurred after the procedure in the following 10 days; however, the patient failed to recover from his hypoxic brain injury and was transferred to the intensive care unit. What is still undetermined is whether the mechanism of the ablation effect is due to the suppression of the trigger or substrate modification. Fundamental improvements in signal processing and filtering technology with new imaging modalities (eg, high-resolution micro-computed tomography [CT], photon-counting CT scanners) are necessary to make Purkinje modulation practical.^38^

## Figures and Tables

**Video 1:** Activation mapping for monomorphic ventricular tachycardia.

**Video 2:** Activation mapping for non-sustained polymorphic ventricular tachycardia with left anterior fascicular exit.
